# Improved Feature-Based Gaze Estimation Using Self-Attention Module and Synthetic Eye Images

**DOI:** 10.3390/s22114026

**Published:** 2022-05-26

**Authors:** Jaekwang Oh, Youngkeun Lee, Jisang Yoo, Soonchul Kwon

**Affiliations:** 1 Department of Electronic Engineering, Kwangwoon University, Seoul 01897, Korea; dhworhkd11@kw.ac.kr (J.O.); yklee1308@kw.ac.kr (Y.L.); jsyoo@kw.ac.kr (J.Y.); 2 Graduate School of Smart Convergence, Kwangwoon Univeristy, Seoul 01897, Korea

**Keywords:** gaze estimation based on feature, eye landmark detection, self-attention, synthetic eye images

## Abstract

Gaze is an excellent indicator and has utility in that it can express interest or intention and the condition of an object. Recent deep-learning methods are mainly appearance-based methods that estimate gaze based on a simple regression from entire face and eye images. However, sometimes, this method does not give satisfactory results for gaze estimations in low-resolution and noisy images obtained in unconstrained real-world settings (e.g., places with severe lighting changes). In this study, we propose a method that estimates gaze by detecting eye region landmarks through a single eye image; and this approach is shown to be competitive with recent appearance-based methods. Our approach acquires rich information by extracting more landmarks and including iris and eye edges, similar to the existing feature-based methods. To acquire strong features even at low resolutions, we used the HRNet backbone network to learn representations of images at various resolutions. Furthermore, we used the self-attention module CBAM to obtain a refined feature map with better spatial information, which enhanced the robustness to noisy inputs, thereby yielding a performance of a 3.18% landmark localization error, a 4% improvement over the existing error and A large number of landmarks were acquired and used as inputs for a lightweight neural network to estimate the gaze. We conducted a within-datasets evaluation on the MPIIGaze, which was obtained in a natural environment and achieved a state-of-the-art performance of 4.32 degrees, a 6% improvement over the existing performance.

## 1. Introduction

Accurately estimating gaze direction plays a major role in applications, such as the analysis of visual attention, research on consumer behavior, augmented reality (AR), and virtual reality (VR). Because inference results are more improved by using deep-learning models than other approaches, they can be applied to advanced technologies, such as autonomous driving [1] and smart glasses [2], and can overcome challenges in the medical field [3]. Using these deep-learning models is quite helpful, but it is difficult to train them due to lighting conditions and insufficient and poor-quality datasets. Moreover, the value of gaze datasets is very expensive and complicated to process. To alleviate this problem, we propose a model that extracts eye features using UnityEyes [4], high-quality synthetic data. An exact position of a feature is obtained from the enhanced model by using a self-attention module. Subsequently, gaze estimation is performed through using high-level eye features, which is less restrictive as it does not utilize complex information, such as full-face information and head poses used for gaze estimation [5,6,7].

Recently, deep-learning-based eye-tracking technology has been developed mainly through appearance-based methods [5,6,7,8,9,10,11] that use eye images or face images. These appearance-based models currently perform particularly well in a controlled, environment in which there are no disturbances, such as noise in an input frame. However, these models have some drawbacks. First, the cost of datasets is very high, and the quality of data has a significant impact on the training of the model. Second, most models are black-box solutions, which pose the challenge of locating and understanding points for improvement. This study reduces the dependency of the feature map, which is difficult to interpret and approaches a feature-based method that can estimate a gaze vector through accurate feature points after acquiring landmarks obtained from an image. Refs. [12,13] used a stacked hourglass model [14] to extract a few eyelid and iris points.

In this study, we reinforced and used an advanced model, called HRNet [15], which shows state-of-the-art performance in the pose estimation task to extract high-quality landmarks. In pixel-wise fields, such as pose estimation and landmark detection, the resolutions and sizes of images have huge impacts on performance. Therefore, we extracted landmarks with a high accuracy by remodeling the model using a self-attention module [16,17,18]. CBAM [18], a self-attention technology, helps to generate a refined feature map that better encodes positional information using channel and spatial attention.

**Figure 1:**
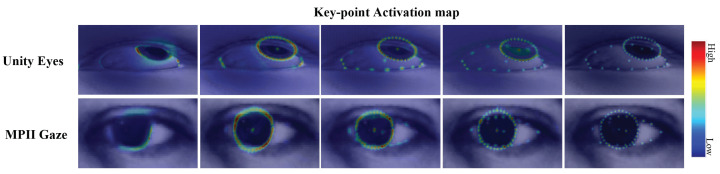
From left to right, the predicted heatmaps are shown as the training epoch increases. The heatmaps have high confidence scores where the landmarks are most likely to be located, and the right bar represents the color space corresponding to the confidence score.

Because we aimed to estimate a gaze vector centered on a landmark extraction, a labeled gaze vector and eye-landmark dataset were essential. However, because gaze data are very expensive and difficult to generate, it is more difficult to obtain a dataset that provides both high-resolution images and landmarks simultaneously. Therefore, UnityEyes, a synthetic eye-image dataset with eye landmarks, was adopted as a training dataset through high-resolution images and an automatic labeling system. The model was trained by processing 32 iris and 16 eyelid points from the eye image obtained by fixing the head pose. Figure 1 shows the predicted heatmaps during the training We evaluated landmark and gaze performance by composing a test set for UnityEyes and performed a gaze performance evaluation using MPIIGaze [11], which has real environment settings.

Our paper is organized as follows. We first summarize related work in Section 2. In Section 3, the proposed gaze estimation method is explained. Section 4 describes the datasets used in the experiments. The experiment results are provided in Section 5. Finally, Section 6 presents our discussion on this study, and Section 7 presents the conclusion.

## 2. Related Work

The gaze estimation method is a research topic of great interest as it has excellent applicabilities to real environments. As it can be applied to various fields, obtaining and creating accurate gaze values and gaze estimations with less constraints are challenging tasks. In this section, we provide a brief overview of the research related to our method. The studies in each subsection are summarized in Table 1, Table 2 and Table 3, respectively.

### 2.1. Feature-Based Method

Feature-based estimation [13,19,20,21,22], a method of gaze estimation, mainly uses unique features that have geometric relationships with the eye. Existing research studies have focused on objects that have a strong visual influences on images.

Roberto et al. [19] used the saliency method to estimate visual gaze behavior and used gaze estimation devices to compensate for errors caused by incorrect calibrations, thereby reducing restrictions caused by user movements. However, the use of these devices increases the error rate as the head moves and interferes with gaze detection.

Some researchers added multiple cameras to compute and focus head movements in multiple directions, extending the influence of eye information and head posture information [20,21]. Head pose has a significant effect on the gaze and requires many restrictions.

To avoid this problem, studies dealing with the static head-pose problem were conducted [13,22] using the convolutional neural network (CNN) model, in which images from a single camera are used to perform gaze estimations based on landmarks as they are less restrictive features. This makes it less difficult to build an experimental environment because there is no need for separate camera calibrations. As only eye images are used for gaze estimations, the dependence of the eye landmark feature vector is increased. After acquiring eye landmarks using a CNN model, a gaze is inferred using support-vector regression (SVR) [23].

Bernard et al. [24] used two gaze maps; one represented the eyeball region and the other represented the iris region. A gaze vector was regressed through the positional relationship between the two gaze maps.

**Table 1:** A summary table of the feature-based gaze estimation method.

Author	Methodology	Highlights	Limitations
Roberto et al. [19]	Saliency method	Fixing the shortcoming of low-quality monocular head and eye trackers	Controlled poses
Manolova et al. [20]	SDM algorithm	Estimating accurate gaze direction based on 3D head positions using a Kinect	Multiple device settings
Lai et al. [21]	CFB and GFB approaches	Integrating CFB and GFB to provide a robust and flexible system	Multiple camera settings
Wood et al. [22]	CNN and 3D head scans	Providing synthetic eye-image datasets with landmarks, a head pose and a gaze direction annotation	Weak to unmodelled occlusions
S. Park et al. [13]	Hourglass network and SVR	Estimating an accurate gaze vector based on eye landmarks in wild settings	Computational costs
Bernard et al. [24]	Capsule network	Utilizing two beat maps where one represents the eyeball and the other represents the iris	Computational costs

**Table 2:** A summary table of the landmark detection.

Author	Methodology	Highlights	Limitations
Toshev et al. [25]	DNN	Introducing a cascade of direct DNN regressors for landmark detection	Overfitting problem
Luvizon et al. [26]	CNN	Using the soft-max function to convert feature maps directly into landmark coordinates in a fully differentiable framework	Limited memory resources
Wei et al. [27]	CPMs	Providing a natural learning-objective function that enforces intermediate supervision to adder the difficulty of vanishing gradients	Enabled for only a single object
Newell et al. [14]	Stacked hourglass	Processing repeated bottom-up and top-down sampling used in conjunction with intermediate supervision	Limited input-image size
Sun et al. [15]	HRNet	Generating highly information-rich feature outputs through a multi-scale feature fusion process	Limited input image size
Yang et al. [28]	TransPose	Introducing a transformer for key-point detections to yield performance improvements through multi-head self-attention	Computationally expensive

**Table 3:** A summary table of the attention mechanism.

Author	Methodology	Highlights	Limitations
Hu et al. [16]	SENet	Providing a novel architectural unit focusing on the channel relationships on feature maps	Lack of information on pixel-wise relationships
J. Park et al. [17]	BAM	Providing a module which infers an attention map along two separate pathways (channel and spatial)	Computational complexity
S. Woo et al. [18]	CBAM	Introducing a lightweight and general module that can be integrated into any CNN architecture	Computational complexity

### 2.2. Landmark Detection

We used a deep-learning-based pose-estimation model as a tool to acquire eye region features. The landmark-detection task includes a key-point detection to detect a skeleton representing the body structure and a facial-landmark detection to extract landmarks in the face; this is a field that requires a large number of datasets depending on the domain. Some models have a direct regression structure based on the deep neural network and have predicted key points [25]. The predicted key-point positions are progressively improved using feedback on the error prediction.

Some researchers proposed a heatmap generation method through using soft-max in a manner that can be fully differentiated [26]. The convolutional pose machine [27] predicts a heatmap with intermediate supervision to prevent vanishing gradients, which are detrimental to deep-learning models. A new network architecture called the stacked hourglass [14] proved that repeated bottom-up and top-down processing with intermediate supervision is an important process for improving performance. A network structure that used high to low sub-networks in parallel is one of the networks that are currently showing the best performance [15]. For a spatially accurate heatmap estimation, high-resolution learning is maintained throughout the entire process; unlike the stack hourglass, it does not use intermediate heatmap supervision, which makes it efficient in terms of complexity and parameters and can generate highly information-rich feature outputs through a multi-scale feature fusion process. Yang et al. [28] introduced a transformer for key-point detections using HRNet as a backbone that extracts a feature map. It causes a performance improvement over existing performance through multi-head self-attention but is computationally demanding.

### 2.3. Attention Mechanism

Attention mechanisms in computer vision aim to selectively focus on the prominent parts of an image to better capture the human visual structure. Several attempts have been made to improve the performance of CNN models in large-scale classification tasks. Residual attention networks improve feature maps through encoder–decoder style attention modules and are very robust to noisy inputs. The attention module reduces the complexity and parameters by dividing calculations into channels and spaces instead of performing calculations in the typical three-dimensional space manner in addition to achieving a significant effect.

The squeeze-and-excitation module [16] proposes an attention module to exploit the relationship between channels. Channel weights are generated through average pooling or max pooling to apply attention to each channel. BAM [17] adds a spatial attention module in addition to the above channel method and places it in the bottleneck to create richer features. CBAM [18] is not only located in each bottleneck of a network but also forms a convolution block to configure the network. In addition, the performance is increased empirically by using the sequential processing method for channel and spatial attention; this method has an empirically better performance than using only the channel unit and has achieved state-of-the-art performance in the classification field.

The self-attention module with such a flexible structure has been applied to many tasks, such as image captioning and visual question answering [29]. The self-attention module is widely used in detection and key-point detection in which spatial information is important [30,31].

## 3. Proposed Method

### 3.1. Overview of Gaze Estimation Based on Landmark Features

**Figure 2:**
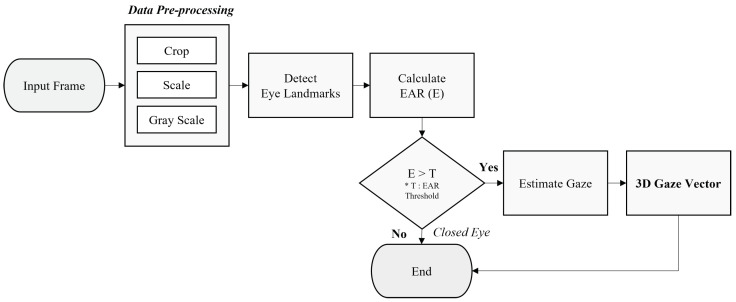
Overall flowchart of our feature-based gaze-estimation system.

In this section, we introduce a network structure and a process for extracting a rich and accurate landmark feature vector from an eye image and then estimating a gaze based on it. A series of procedures for estimating a proposed gaze is shown in Figure 2. Eye images can simply be acquired from a single camera. If a frame contains a full-face image, the frame must be cropped to a 160×96 sized image centered on the eye area using the face detection algorithm [32]. The image is converted into black and white image for simple processing. This can enhance the performance output of the infrared camera.

To obtain eye-feature vectors from processed images, we selected HRNet as a baseline model that can generate feature maps containing rich information through fusions with various feature maps while maintaining a high image resolution. HRNet showed the best performance in the key-point detection task, proving its utility. We modified HRNet by additionally using the self-attention module CBAM. Channel-wise and spatial-wise weights were applied to infer the most important channels in the 3D feature map and most important spatial points in the channels. Section 5.1 shows that the proposed model achieved higher landmark accuracy than models in previous studies.

The EAR [33] threshold (*T*), which can be different for each individual, was set using the initial 30 input frames. The EAR ratio value (*E*) was calculated for each frame; if the calculated EAR ratio value was less than the threshold value, it was judged that there was no need to estimate gaze because the eyes were closed. By reducing false-positive errors, it was possible to proceed with a gaze estimation that had a computational advantage. In some cases, 3D gaze regressions use SVR, but we proceeded by constructing an optimal MLP. The architecture configurations of these models have the advantage of being able to proceed one step when learning.

The most important task of our proposed method was to acquire a high-level landmark feature that affects the EAR ratio and gaze. Before training the model, we were faced with the problem of a lack of a dataset, which is a chronic problem of deep-learning models, adversely affects their training, and can result in over-fitting. Landmark datasets are especially expensive, and only a few datasets include both a gaze and a landmark. To avoid this problem, we used a large set of UnityEyes synthetic data for training the dataset. UnityEyes synthetic data is a dataset that includes annotations, such as rich eye landmarks and gazes, by modeling a 3D eyeball based on an actual eye shape created by using the game engine Unity. The models [4] trained with this synthetic dataset showed good performances and had a lot of information and high resolutions; therefore, they are very suitable for processing and applications.

### 3.2. Architecture of Proposed Landmark-Detection Model

We used a feature vector for gazes with large amounts of eye landmarks obtained through the model from the input frame that contains eye information from a single camera. To increase the gaze accuracy, it was important to generate a high-level feature, and we set the advancement of the model that extracted a heatmap output most similar to the correct answer as the main goal of this study. Previous studies [12,13] that used eye landmarks as features mainly adopted [14] the production of feature outputs. However, because the feature map is restored through decoding after passing forward from high resolution to low resolution, it is weak in expression learning at a high resolution. Because our model requires extracting more eye landmarks from small-sized eye images than previous studies [13], feature learning at high resolution, which has high sensitivity to positional information in an image space, was necessary. Therefore, we adopted HRNet, which maintains multi-resolution learning (including a high-resolution), as a baseline model.

The basic structure of HRNet consists of 4 steps and 4 stages. Each step creates a feature that doubles the number of channels with half the resolution of the previous step. Each stage consists of a residual block and an exchange unit, and the feature map of each step is processed in parallel. The exchange unit is an information exchange process through fusion between feature maps of each step through fusion, and the second, third, and fourth stages have one, four, and three units, respectively. At the end of each stage, there are feature fusion and transition processes that increase a step by generating a feature map that is half the previous size. Fusion between multi-scale features includes an up-sampling process that uses 1×1 convolution, a nearest-neighbor interpolation in the bottom-up path, and a down-sampling process that uses several 3×3 convolution blocks with strides of 2 in the top-down path.

(1) $$\begin{array}{c} input=\left\{X_{1},X_{2},...X_{r}\right\}\;output=\left\{Y_{1},Y_{2},...Y_{r}\right\}\\ Y_{k}=\sum_{i=1}^{r}F\left(X_{i},k\right)\;,\;F\left(X_{i},k\right)=\left\{\begin{array}{cc} identify\;connection, & \mathrm{if}\;i=k\\ up\;sampling, & \mathrm{if}\;i<k\;\;(i,k\leq r)\\ down\;sampling, & \mathrm{if}\;i>k \end{array}\right. \end{array}$$

Equation (1) describes the feature fusion process. For input $\left\{X_{1},X_{2},...X_{r}\right\}$ of different resolutions, output features $\left\{Y_{1},Y_{2},...Y_{r}\right\}$ are generated through an element-wise summation of features after down-sampling and up-sampling. *r* represents resolution numbers; if *r* is the same, the widths and resolutions of the input and output are the same. At the end of the 4th stage, all step information is concatenated to create the feature block $F_{b}\left\{[Y_{1}^{4};Y_{2}^{4};Y_{3}^{4};Y_{4}^{4}]\right\}$ and to head to the prediction head.

To solve the problem of the typically acquired eye image having a small resolution, we introduced an additional residual block layer composed of a 3×3 convolution to the model to create feature ($F_{o}$) of the origin resolution that stores the information of the largest resolution. Through the summation of $F_{o}$ and up-sampled $F_{b}$, more spatially accurate features are created.

**Figure 3:**
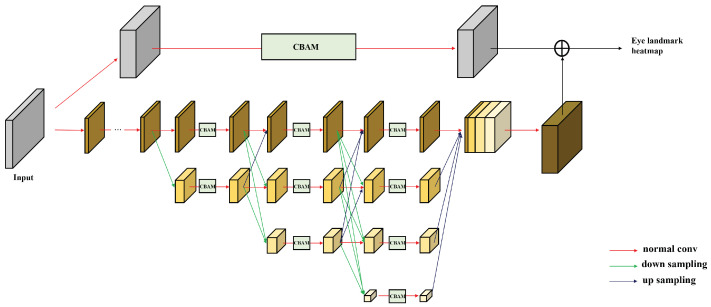
Our landmark-detection network architecture used to extract feature map.

Because the heat map, which is the final result of the network, requires accurate spatial information for each channel, we applied CBAM, a self-attention technique, to the normal residual and convolution blocks of each stage. Architecture of the modified network is illustrated in Figure 3. These techniques (adding the residual CBAM layer and applying CBAM to all stages of the residual block) improved the landmark-detection performance, which is described in Section 5.1.

### 3.3. Network Engineering with the Self-Attention Module CBAM

**Figure 4:**
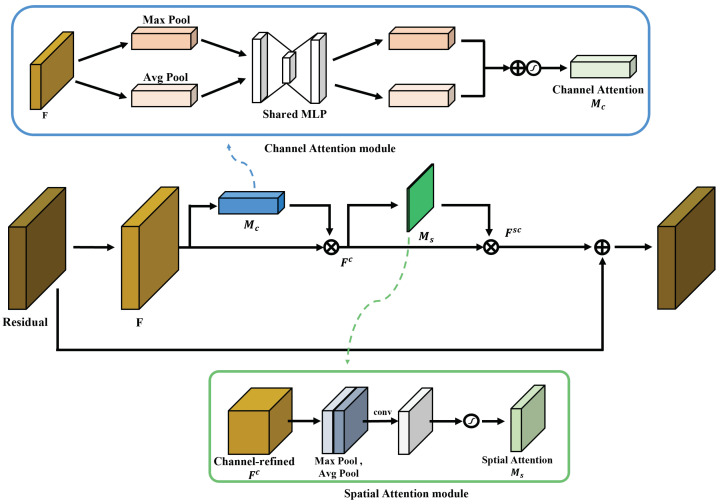
Convolutional block attention module (CBAM) architecture in residual block.

Attention mechanisms have been widely used for feature selection using multi-modal relationships. Refining the feature maps using attention module helps the network and causes it to perform well and become robust to noisy inputs. Based on empirical results, such as those in [16,17], the CBAM self-attention module has developed rapidly and showed higher accuracy than existing modules in the image classification task through various structure and processing experiments. We judged that the positional information of the refined feature would improve the performance; therefore, we applied the residual block of the network by replacing the CBAM block. The architecture of CBAM is illustrated in Figure 4.

CBAM adds two sub-networks that consist of channel attention and spatial attention networks to the basic residual block. Feature $F\in R^{\;C\times H\times W}$ is generated through a 3×3 convolution of the residual, which is the output of the previous block. *F* goes through the channel attention and spatial attention networks sequentially. First, in the case of channel attention, the two types of channel-wise pooling, that is, max pooling and average pooling, are performed to obtain weight parameters for channels. Feature vectors $F_{max}\in R^{\;C\times 1\times 1}$ and $F_{avg}\in R^{\;C\times 1\times 1}$, generated through pixel-wise pooling, share an MLP that has a bottleneck structure with the advantages of parameter reduction and generalization and are merged using element-wise summation. Finally, the product is normalized using sigmoid function to obtain the meaningful weights $M_{c}\left(F\right)\in R^{\;C\times 1\times 1}$ and generate $F^{c}\in R^{\;C\times H\times W}$ by multiplying $M_{c}\left(F\right)$ and *F*. The above process is described by using Equation (2).

(2) $$\begin{array}{c} M_{c}\left(F\right)\;=\;F_{sigmoid}\left(MLP\left(F_{max}\right)+MLP\left(F_{avg}\right)\right),\\ F^{c}\;=\;M_{c}\left(F\right)⨂F \end{array}$$

Subsequently, using the channel-refined feature ($F^{c}$) as an input, $M_{s}\left(F^{c}\right)$ is generated through the spatial attention module.

(3) $$\begin{array}{c} M_{s}\left(F^{c}\right)\;=\;F_{sigmoid}\left(Conv_{7\times 7}\left(\left[F_{max};F_{avg}\right]\right)\right),\\ F^{sc}\;=\;M_{c}\left(F^{c}\right)⨂F^{c},\\ output=Residual⨁F \end{array}$$

In Equation (3), spatial weight feature $M_{s}\left(F\right)\in R^{\;1\times H\times W}$ is made by using sequential process pooling, concatenation, 7×7 convolution and normalizing using with sigmoid function, then $F^{sc}\in R^{\;C\times H\times W}$ is merged by multiplying $M_{s}\left(F^{c}\right)\in R^{\;1\times H\times W}$ and $F^{c}$. The output of blocks that are merged by using the element-summation residual and $F^{sc}$ is refined with a focus on ‘what’ and ‘where’, respectively. Because we applied this module to the residual block of the processing stage in parallel, the output at each stage contains very rich information and encodes channel information at each pixel over all spatial locations due to attention and fusion.

We applied the CBAM module of the additional residual layer and additionally applied the CBAM module to all steps of the stage. We showed performance improvement through the normalized mean error (NME) value, which is a key-point-detection performance value. Detailed outcome indicators are described in Section 5.

### 3.4. Gaze-Estimation-Based Eye Landmarks with EAR

We estimated gaze vectors based on an eye feature that consists of a total of 50 eye landmarks (1 from an eye center, 1 from an iris center, 16 from an eyelid, and 32 from an iris). We extracted high-accuracy eye landmark localization while optimizing and improving the network. As the quality of the landmark extracted by the network improved, the gaze regression performance also improved empirically. Existing feature-based studies [13,34] mainly used the SVR for gaze regressions. We empirically confirmed that the difference between the SVR and multi-layer perceptron (MLP) performance is very small and that the MLP performance is relatively good. The MLP simply contains two hidden layers and uses Leaky ReLU [35] as an activation function. In addition, when the MLP is used, there is the advantage that landmark detection and gaze estimation are possible in one-stage training. The MLP contains two hidden layers and uses Leaky ReLU as an activation function. The co-ordinates used as the inputs are normalized to the distance between the eye endpoints, and all eye points are translated with respect to the eye center coordinates.

**Figure 5:**
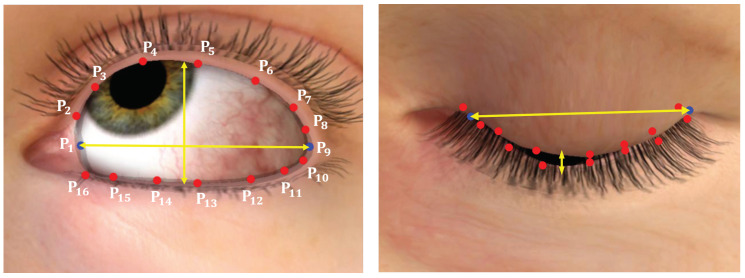
EAR was calculated through the displayed landmark coordinates (from $P_{1}$ to $P_{16}$). The blue dots are used to represent the width of the eye and the red the height. The eye on the right is in a state at which there is no need to estimate the gaze.

To reduce false positives and increase efficiency, we utilized an EAR value. The EAR value was calculated to decide whether an eye was closed or not using 16 eyelid points. We introduced a new EAR metric based on a method that uses 6 points because we could obtain richer, high-quality eyelid points. Figure 5 shows the measured lengths of an eye using images that include a closed eye. We measured the horizontal length through the $p_{1}$ and $p_{9}$ points out of a total of 16 points $\left\{p_{1},p_{2},...p_{16}\right\}$, and the average value of the remaining seven pairs of points $\left\{\left(p_{2},p_{16}\right),\left(p_{3},p_{15}\right)...\left(p_{8},p_{10}\right)\right\}$ was defined as the vertical length. The EAR was calculated using Equation (4).

(4) $$\begin{array}{c} EAR=\frac{\sum_{n=1}^{7}{\|p_{n+1}-p_{17-n}\|}_{1}}{{7\|p_{1}-p_{9}\|}_{1}} \end{array}$$

Because the EAR varies considerably from user to user, we set the EAR threshold (*T*) to half the median value after receiving the EARs for the initial 30 frames’ inputs. Then, if the measured EAR was smaller than *T*, the network did not estimate gaze.

### 3.5. Learning

Two losses, a landmark loss and a gaze loss, were required for the training of our proposed network. The method of regressing the heatmap, which is the probability of the existence of each feature point using a CNN model, has fewer parameters than the method of directly regressing the feature point coordinates and can avoid the problem of over-fitting. However, it is difficult to precisely detect units below the decimal point because heatmap regression acquires integer co-ordinates through an arg-max operation in the process of converting heatmap into coordinates. We used integral regression [36] to properly compensate for the above two shortcomings. The integral regression module removes negative values by applying AB ReLU operation to the heatmap and divides the operation by the total sum to normalize it. As shown in Equation (5), all values of $\hat{H}$ are between 0 and 1 and the total sum becomes 1; therefore, it is defined as a probability distribution. Subsequently, the co-ordinates of each feature point in the heatmap can be obtained through the expected value calculation.

(5) $$\begin{array}{c} \hat{H_{c}}\left(x,y\right)=\frac{F_{ReLU}\left(H_{c}\left(x,y\right)\right)}{\sum_{i}\sum_{j}(H_{c}\left(i,j\right)}\\ predicted\;coordinates=\left\{\begin{array}{c} x_{c}=\sum_{i}\sum_{j}i\hat{H_{c}}\left(i,j\right)\\ y_{c}=\sum_{i}\sum_{j}j\hat{H_{c}}\left(i,j\right) \end{array}\right. \end{array}$$

Therefore, the final landmark cost function consists of the mean squared error (MSE) loss between the output and the ground-truth heatmap, the L1 loss of the ground-truth co-ordinate and the co-ordinates obtained by using the expected value operation. $\;H^{{}^{\prime }}$ is the predicted heatmap, H is the ground-truth heatmap, $\left(x^{{}^{\prime }},y^{{}^{\prime }}\right)$ is the co-ordinate predicted through the integral module, and (x,y) is the ground-truth co-ordinate.

(6) $$\begin{array}{c} Loss_{heatmap}=\sum_{i}\sum_{x}\sum_{y}\|H_{i}^{{}^{\prime }}\left(x,y\right)-H_{i}{\left(x,y\right)\|}_{2}^{2}\;,Loss_{coordinates}=\sum_{i}{\left\|\left(x^{{}^{\prime }},y^{{}^{\prime }}\right)-\left(x,y\right)\right\|}_{1}\\ Loss_{landmark}=Loss_{heatmap}+Loss_{coordinates} \end{array}$$

**Figure 6:**
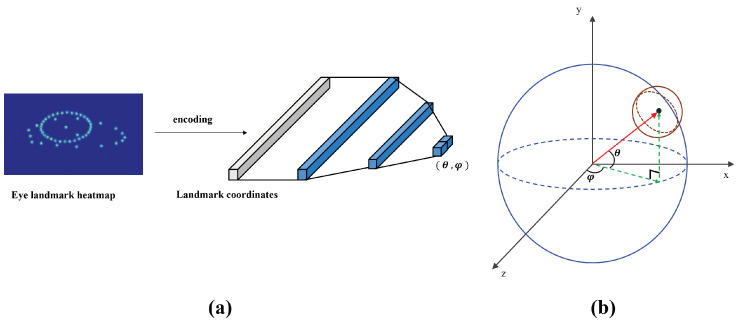
(**a**) illustrates the simple network architecture for gaze estimation and (**b**) shows the relationship between the pupil and the eyeball. Gray embedding vector encode the landmarks coordinates. Gaze vector (red) can be explained through a pitch (θ) and yaw (φ).

To compare each gaze performance, experiments were conducted using several methods. There are two frequently used methods of gaze regression. The first is a method of directly regressing a 3D vector and the second is a method of encoding a 3D normal vector into 2D space pitch (θ) and yaw (φ) regression. The pitch and yaw are the angles between the pupil and the eyeball, which can explain the positional relationship. The positional relationship between an eyeball and a pupil is illustrated in Figure 6. We found that the generalization was better when a 2D angle vector was encoded empirically and cosine distance loss and MSE were used as cost functions, and the best performance was obtained in MSE. (Pitch, yaw), that is, $\left(\theta^{{}^{\prime }},\varphi^{{}^{\prime }}\right)$, is the predicted 2D gaze and (θ,φ) is the ground-truth 2D gaze.

(7) $$\begin{array}{c} pitch\left(\theta \right)=\arcsin\left(y\right),\;yaw\left(\varphi \right)=\arctan\left(\frac{x}{z}\right)\\ Loss_{gaze}={\left\|\left(\theta^{{}^{\prime }},\varphi^{{}^{\prime }}\right)-\left(\theta ,\varphi \right)\right\|}_{2}^{2} \end{array}$$

We trained our model using a UnityEyes dataset that consists of 80,000 images, and each validation and test used 10,000 images. We used black and white 1×160×96 images and set batch size to 16. We used the Adam optimizer. The learning schedule followed the settings in [15]. We used pre-trained data on ImageNet. The base learning rate was set as 4 × 10${}^{-4}$ and decreased by every 25 epochs. Specifications of the PC used in the experiment were an Intel Core i9-11900K, 3.5 GHz CPU, and NVIDIA RTX 3090 GPU with 24 GB of memory for training.

## 4. Description of the Dataset

**Figure 7:**
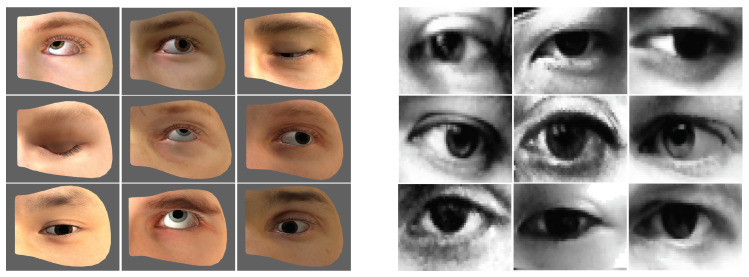
Samples from two datasets: left is UnityEyes and right is MPIIGaze.

This section describes the dataset used for network training and evaluation. Figure 7 shows the original forms of the utilized datasets.

### 4.1. UnityEyes

In a real-world setting, datasets for gaze estimation are very expensive to acquire, do not support eye landmarks, or are very poor; therefore, they are inadequate for training a network. We selected UnityEyes synthetic datasets for training to solve the above problem. UnityEyes creates an eye model by manipulating several parameters using the Unity game engine and provides high-resolution 2D images from the camera position, high-quality 3D eye coordinates, and a 3D gaze vector. We also processed rich annotations and utilized them for network learning. Previous studies [4,13] showed good performance using synthetic datasets.

**Figure 8:**
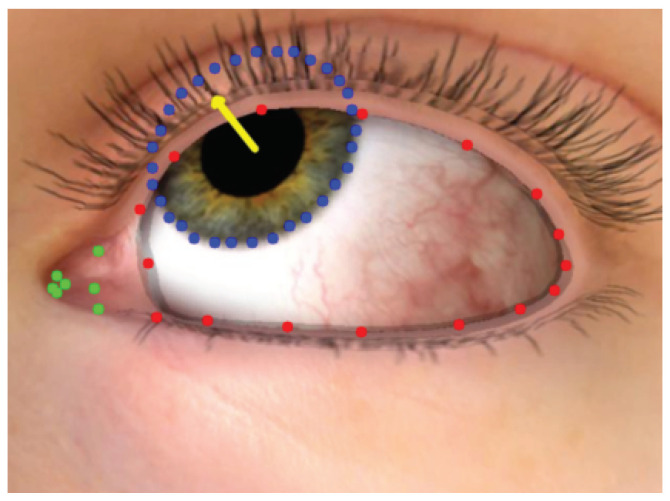
An annotated sample from UnityEyes. The red, green, and blue points are 16 eye edges, 7 caruncles, and 32 iris edges, respectively. The yellow arrow represents the 3D gaze direction.

An eye landmark provided in UnityEyes is presented in Figure 8. A total of 53 eye landmarks consisting of 16 eye edges, 7 caruncles, and 32 iris edges were used. We used all the labeled eye and iris edges while ignoring the caruncles because it was judged that they would have no effect on gaze. Subsequently, the eyes and iris centers, which were mean values of all the eyes and iris edges, were added to configure the ground-truth with a total of 50. It was possible to create a resolution of 640×480 up to 4K, and we cropped an 800×600 image to a 160×96 size.

### 4.2. MPIIGaze

The MPIIGaze datasets were recorded using a laptop for several months over the daily lives of 15 experimental participants. The datasets were representative evaluations and very suitable for judging the performance of networks in uncontrolled settings. They were proposed in 2015 and include a head pose vector, gaze vector, full-face image, and 60×36 normalized image required for evaluations. The datasets also provide eye landmark co-ordinates of six eye edges and one iris center but do not provide enough for within-dataset leave-one-person-out evaluations [11]. That is, the proposed network was trained using the data of 14 participants (3000 images consisting of left and right eyes) and then validated on the data of an excluded person. Thus, we made a labeling tool for rich eye landmarks as one is required by a neural network that is robust against noise.

**Figure 9:**
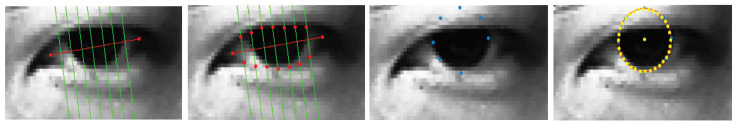
From left to right presents the sequential labeling process. The red and green lines and blue dots are tools for landmark coordinates, and the red and yellow dots represent annotations. Outputs are comprised of 16 eye edges, 32 iris edges, 1 eye center, and 1 iris center.

Figure 9 illustrates an overview of this labeling tool. First, when a user draws a point on both endpoints of an eye, a line connecting the two points and straight lines dividing the line into eight equal parts are created. Subsequently, the remaining eye-edge co-ordinates are obtained by dotting the points at which the eight straight lines and eye region overlap. At this time, a correction effect is applied so that the drawn points lie on the straight lines. When a total of 16 eye edges are completed, the user specifies an elliptical area that can include the iris area. When eight dots are obtained, an ellipse is generated that best contains the iris through the RANSAC algorithm [37]. Users obtain 32 iris edges spaced at regular intervals from the ellipse. The datasets and code on 12 April 2022 are available to the public: https://github.com/OhJaeKwang/Eye_Region_Labeling.

## 5. Experiments

In this section, we describe the experiments conducted in this study. The experiments included an evaluation considering the performance of landmark detection and gaze estimation with respect to MPIIGaze (45K) and UnityEyes (10K). The NME and mean angle error (MAE) were adopted as metrics for evaluation.

### 5.1. Landmark-Detection Accuracy

There are several metrics for evaluating the accuracies of landmarks, but we adopted the NME [38], which is main metric used in facial landmark detection and is the most relevant among them. The NME represents the average Euclidean distance between an estimated landmark position ($P^{{}^{\prime }}$) and a corresponding ground truth (*P*). The NME is calculated using Equation (8), where *N* is the number of images, *L* is the number of landmarks, and *d* is defined as average eye width of a test set for the normalization factor.

(8) $$\begin{array}{c} NME=\frac{1}{N}\sum_{i=1}^{N}\frac{\sum_{j=1}^{L}\|P_{i,j}^{{}^{\prime }}-P_{i,j}{\|}_{2}^{2}}{L\times d} \end{array}$$

**Figure 10:**
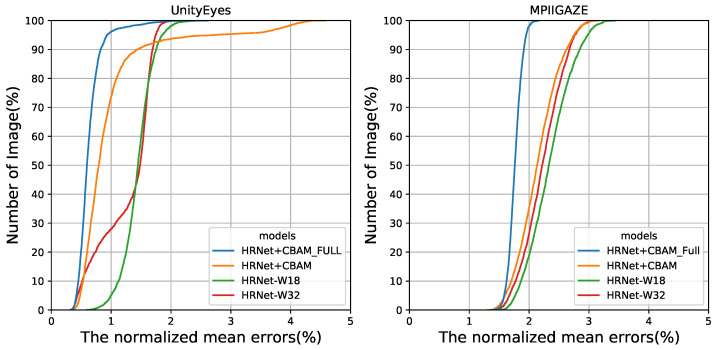
Comparisons of the cumulative error distribution curves of the test datasets. We compared our method with baseline approaches (HRNet). HRNet+CBAM and HRNet+CBAM_FULL denote adding a residual CBAM layer and applying CBAM to all stages of the residual blocks, respectively.

We compared our approach to a baseline model. Two approaches were introduced as follows. The first added a CBAM residual layer and the second applied CBAM to convolution blocks of all stages. We used the model parameters trained on UnityEyes. The landmark detection results of our approaches and the baseline model (HRNet-W18) with respect to MPIIGaze and UnityEyes are shown in Table 4. HRNet-w18 and HRNet-w32 are lightweight models of HRNet, and 18 and 32 indicated the channel multiples of the last stage. In the results, each approach showed a better performance than the existing model, and the final model achieved an approximately 4% higher NME score compared to HRNet-W18 on all datasets. Graphs showing the ratios of the test sets according to the NME value are presented in Figure 10. Similarly, the AUC [39] value (the area under the curve) demonstrated that the two approaches using self-attention improved performance.

Because the MPIIGaze dataset before pre-processing consisted of a very low resolution of 60×36, we interpolated it with a 160×96 dataset and processed the result; we judged that the performance with respect to MPIIGaze was inferior to UnityEyes due to noise generated during this process, problems of poor quality, and reliability of labeling.

### 5.2. Gaze Estimation Accuracy

**Figure 11:**
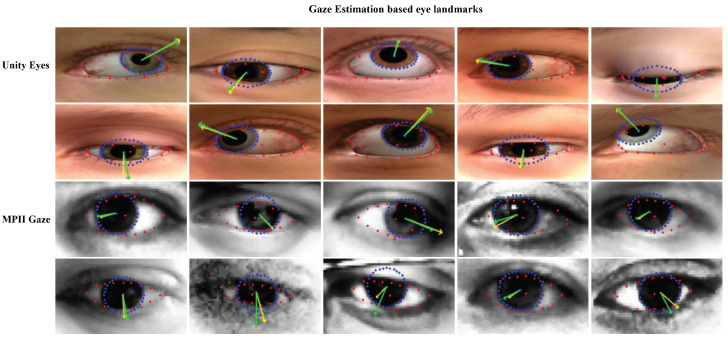
Results of our gaze estimation system with respect to UnityEyes and MPIIGaze test sets. The red, blue points represent the iris edge and the eye edges, respectively. Ground-truth gaze is represented by green arrows and predicted gaze is represented by yellow arrows.

Our method, which showed the best landmark performance, achieved an angle error of 1.7 for 10,000 UnityEyes test sets. Subsequently, we compared various systems for within-dataset evaluation (leave-one-person-out strategy) to the MPIIGaze dataset using an MAE that represents the differences in the angles of two unit vectors. The results of the models evaluated using the MPIIGaze dataset, usage techniques, and information used as inputs are included in Table 5. Our method achieved a competitive degree of error in the above experiment. Fine-tuning the model parameters pre-trained on UnityEyes using MPIIGaze improved the performance by approximately 6.80% (from 4.64${}^{\circ }$ to 4.32${}^{\circ }$), and our approach surpassed the baseline method (from 4.60${}^{\circ }$ to 4.32${}^{\circ }$). We improved the performance by approximately 6.04% compared to the baseline model. Additionally, unlike the appearance method, our method was less constrained by registration conditions and had better usability in that we could create high-level landmarks. This result showed that the performance improvement of landmark detection had an effect on gaze regression. We show the qualitative predictions of our gaze estimation system with respect to UnityEyes and MPIIGaze in Figure 11. It was observed that it acquired high-level features even for noisy MPIIGaze data and had good gaze accuracy.

## 6. Discussion

Applications that utilize gaze information practically and visually provide novelty and satisfaction to users, so it is essential to improve the accuracy of predicted information. To achieve a performance improvement, we conducted a study by adapting the feature-based method, which is better for generalizing than the appearance-based method. In prior work, features were usually hand-crafted for gaze using image-processing and model-fitting techniques. However, because these approaches make assumptions about geometry, such as the 3D eyeball and 3D head coordinates, they are sensitive to noise in uncontrolled real-world images.

In this study, we proposed a gaze estimation method using a more accurate and detailed eye region where eye landmarks represent the locations of the iris and eyelid. We used the UnityEyes dataset, which has high quality annotation that helped the representation learning of our network.

Since we assumed that the accuracy of gaze estimation increases as the confidence of the landmarks intended to be used as features increases, we tried to develop an advanced landmark-detection model. We also assumed that the feature map of the layer should represent meaningful location information and proposed a method combining the self-attention module with the model. The first results suggested that adding the self-attention module improves the inference accuracy. In particular, the best performance improvements were seen with negligible overheads when the module was applied to all layers. Moreover, since the inference accuracy for low-quality MPIIGaze had increased, it was shown to be robust to the noise of the input data. Then, we were able to confirm that the performance of landmark and gaze were proportional through considering the second result. We obtained a meaningful study, but there difficulties were encountered during the study.

We had to train the models on the real-world MPIIGaze dataset for the evaluation. Unavoidably, in order for our network to learn, we needed to take landmark annotations unconditionally. However, MPIIGaze didn’t have as many as we needed. Consequently, we made a labeling tool and labeled MPIIGaze (45K) using it. An unsupervised domain adaptation [40] can solve this limitation. It does not require annotations on the target domain and is used for only feature training for a target. Using generative adversarial networks (GAN), the method for a fusion between datasets from different domains might help a model to perform transfer learning well [41]. To alleviate the limitation, the hope is that our work will apply these skills to our method.

## 7. Conclusions

In this study, we proposed a feature-based gaze system that achieved a higher accuracy than existing models trained on the same datasets by introducing a network to extract high-level landmarks. Contrary to the existing methods, we predicted a heatmap with richer representations from the transferred multi-scale features using HRNet to obtain more accurate and more spatially precise eye features. Moreover, we achieved the best performance improvement by applying a self-attention module that emphasized meaningful features in the principal dimensions, which were the channel and spatial axes of the feature map, in addition to achieving efficient computational and parameter overheads. Using UnityEyes, which supports a high-level annotation and a high resolution, we were able to extract more and greater landmarks, and these richer landmarks resulted in a competitive gaze accuracy for a within-dataset evaluation with respect to MPIIGaze. Additionally, our method had less restrictive registration conditions and great utility in providing landmarks.

During the experiment, we found that the transfer learning of the model through various real-world gaze datasets was superior to the results of the model trained with only UnityEyes. However, our model required numerous landmark annotations, and there was no dataset that satisfied this requirement. To solve this problem, we used a labeling tool in this study. However, in the next study, we plan to apply the unsupervised domain adaptation technique to optimize the model using UnityEyes and real-environment datasets without using a key-point annotation simultaneously.

## Figures and Tables

**Table 4:** The approaches that applied a CBAM module improved the quantitative metric.

Method	UnityEyes		MPIIGaze	
	NME (%) ↓	AUC ↑	NME (%) ↓	AUC ↑
HRNet-W18	7.21	75.95	11.71	60.93
HRNet-W32	6.69	78.79	11.13	62.91
HRNet + CBAM	4.95	83.49	10.72	64.20
HRNet + CBAM_FULL	3.18	89.39	8.82	70.59

**Table 5:** Comparing the MAE, representation, and registration of several methods evaluated using MPIIGaze. (*: baseline method).

Method	MAE (°)	Representation	Registration
RF [10]	7.99${}^{\circ }$	Appearance	Eyes and head pose
Mnist [11]	6.30${}^{\circ }$	Appearance	Single eye
GazeNet [8]	5.83${}^{\circ }$	Appearance	Single eye
AR-Net [9]	5.65${}^{\circ }$	Appearance	Eyes
ARE-Net [9]	5.02${}^{\circ }$	Appearance	Eyes
* S. Park et al. [13]	4.60${}^{\circ }$	Feature and gaze regression network	Single eye
S. Park et al. [12]	4.50${}^{\circ }$	Appearance	Single eye
FARE-Net [6]	4.41${}^{\circ }$	Appearance	Face, eyes
Ours	4.32${}^{\circ }$	Feature and gaze regression network	Single eye

## Data Availability

Not applicable.
